# Characterization of Post–COVID-19 Definitions and Clinical Coding Practices: Longitudinal Study

**DOI:** 10.2196/53445

**Published:** 2024-05-03

**Authors:** Monika Maripuri, Andrew Dey, Jacqueline Honerlaw, Chuan Hong, Yuk-Lam Ho, Vidisha Tanukonda, Alicia W Chen, Vidul Ayakulangara Panickan, Xuan Wang, Harrison G Zhang, Doris Yang, Malarkodi Jebathilagam Samayamuthu, Michele Morris, Shyam Visweswaran, Brendin Beaulieu-Jones, Rachel Ramoni, Sumitra Muralidhar, J Michael Gaziano, Katherine Liao, Zongqi Xia, Gabriel A Brat, Tianxi Cai, Kelly Cho

**Affiliations:** 1 Veterans Affairs Boston Healthcare System Boston, MA United States; 2 Department of Biomedical Informatics Harvard Medical School Boston, MA United States; 3 Department of Population Health Sciences University of Utah Salt Lake City, UT United States; 4 Department of Biomedical Informatics University of Pittsburgh Pittsburgh, PA United States; 5 Office of Research and Development US Department of Veterans Affairs Washington, DC United States; 6 Division of Aging, Department of Medicine Mass General Brigham Harvard Medical School Boston, MA United States; 7 Division of Rheumatology, Inflammation, and Immunity Brigham and Women's Hospital Boston, MA United States; 8 Department of Neurology University of Pittsburgh Pittsburgh, PA United States; 9 Department of Surgery Beth Israel Deaconess Medical Center Boston, MA United States

**Keywords:** veterans, long COVID-19, postacute sequelae of SARS-CoV-2, PASC, International Classification of Diseases, U09.9 ICD-10 code, algorithm validation, chart review, electronic health records, COVID-19

## Abstract

**Background:**

Post–COVID-19 condition (colloquially known as “long COVID-19”) characterized as postacute sequelae of SARS-CoV-2 has no universal clinical case definition. Recent efforts have focused on understanding long COVID-19 symptoms, and electronic health record (EHR) data provide a unique resource for understanding this condition. The introduction of the *International Classification of Diseases, Tenth Revision* (*ICD-10*) code U09.9 for “Post COVID-19 condition, unspecified” to identify patients with long COVID-19 has provided a method of evaluating this condition in EHRs; however, the accuracy of this code is unclear.

**Objective:**

This study aimed to characterize the utility and accuracy of the U09.9 code across 3 health care systems—the Veterans Health Administration, the Beth Israel Deaconess Medical Center, and the University of Pittsburgh Medical Center—against patients identified with long COVID-19 via a chart review by operationalizing the World Health Organization (WHO) and Centers for Disease Control and Prevention (CDC) definitions.

**Methods:**

Patients who were COVID-19 positive with either a U07.1 ICD-10 code or positive polymerase chain reaction test within these health care systems were identified for chart review. Among this cohort, we sampled patients based on two approaches: (1) with a U09.9 code and (2) without a U09.9 code but with a new onset long COVID-19–related ICD-10 code, which allows us to assess the sensitivity of the U09.9 code. To operationalize the long COVID-19 definition based on health agency guidelines, symptoms were grouped into a “core” cluster of 11 commonly reported symptoms among patients with long COVID-19 and an extended cluster that captured all other symptoms by disease domain. Patients having ≥2 symptoms persisting for ≥60 days that were new onset after their COVID-19 infection, with ≥1 symptom in the core cluster, were labeled as having long COVID-19 per chart review. The code’s performance was compared across 3 health care systems and across different time periods of the pandemic.

**Results:**

Overall, 900 patient charts were reviewed across 3 health care systems. The prevalence of long COVID-19 among the cohort with the U09.9 ICD-10 code based on the operationalized WHO definition was between 23.2% and 62.4% across these health care systems. We also evaluated a less stringent version of the WHO definition and the CDC definition and observed an increase in the prevalence of long COVID-19 at all 3 health care systems.

**Conclusions:**

This is one of the first studies to evaluate the U09.9 code against a clinical case definition for long COVID-19, as well as the first to apply this definition to EHR data using a chart review approach on a nationwide cohort across multiple health care systems. This chart review approach can be implemented at other EHR systems to further evaluate the utility and performance of the U09.9 code.

## Introduction

Characterizing the public health burden of postacute sequelae of SARS-CoV-2, also known as post–COVID-19 condition or colloquially as long COVID-19, has been difficult, given that multiple clinical case definitions have been proposed by various international health agencies [1-3]. While the exact components of these definitions vary, they share some common underlying features such as the development of symptoms that are new onset after a COVID-19 infection and the persistence of new onset symptoms for a duration of time after acute infection period. Electronic health records (EHRs) provide a uniquely rich resource for studying this condition at scale, and there have been multiple efforts to describe long COVID-19 symptoms and estimate prevalence in various EHR systems [4-9].

Specifically, the introduction of the *International Classification of Diseases, Tenth Revision* (*ICD-10*) code U09.9 for “Post COVID-19 condition, unspecified” has provided an alternative method of evaluating this condition in EHRs, and its use has been described in various health care systems [10-13]. However, there is no universal diagnostic guideline for defining long COVID-19, and thus, there is no standard guideline for assigning U09.9. The use of the U09.9 code and its accuracy in identifying long COVID-19 have not yet been evaluated against any existing clinical case definitions in a multicenter setting. Clinical coding of long COVID-19 has the potential for misclassification, given the heterogeneity and ambiguity around the definition of long COVID-19 [14].

This study aims (1) to characterize the use of *ICD-10* code U09.9 across 3 health care systems and (2) to evaluate the accuracy of the U09.9 code against patients identified with long COVID-19 via chart review.

## Methods

### Data Sources and Study Cohort

The Consortium for Clinical Characterization of COVID-19 by EHR (4CE) is an international consortium for data-driven studies on the COVID-19 pandemic [15]. Three health care systems from the 4CE contributed chart review results for this study, namely, the national Veterans Health Administration (VHA), the Beth Israel Deaconess Medical Center (BIDMC), and the University of Pittsburgh Medical Center (UPMC). Over 15 million patients are collectively provided care across all 3 health care systems [16-18]. The VHA is the largest integrated health care system in the United States with 171 medical centers throughout the country [16]. The BIDMC is an academic medical center that is part of Beth Israel Lahey health care system located in Boston, and the UPMC is a Pittsburgh-based health care system with 40 hospitals across Pennsylvania [17,18].

**Figure 1:**
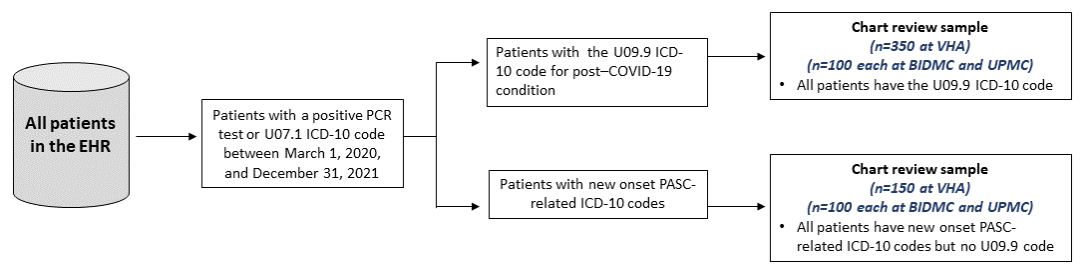
Patient sampling strategy for chart review. BIDMC: Beth Israel Deaconess Medical Center; EHR: electronic health record; ICD-10: International Classification of Diseases, Tenth Revision; PASC: postacute sequelae SARS-CoV-2; PCR: polymerase chain reaction; UPMC: University of Pittsburg Medical Center; VHA: Veterans Health Administration.

EHR data from the 3 health care systems were used to identify patients who were COVID-19 positive, define patient characteristics, and obtain clinical notes for chart review. The sampling strategy for our chart review is described in Figure 1. Patients who had their first incidence of COVID-19 diagnosis reported within the participating health care systems’ EHR with either a U07.1 ICD-10 code for “COVID-19” or a positive polymerase chain reaction test performed between March 1, 2020, and December 31, 2021, were identified for chart review. From this COVID-19–positive cohort, we then sampled patients based on two approaches: (1) the presence of the U09.9 ICD-10 code, which was first introduced in the United States in October 2021, or (2) the presence of at least 1 new onset long COVID-19–related ICD-10 code if the patient did not have a U09.9 code. These long COVID-19–related ICD-10 codes were selected to enrich the chart review sample for patients who may potentially have long COVID-19. At the VHA, we further sampled patients from 2 time periods: those who were COVID-19 positive before September 1, 2021 (pre-U09.9 period), and those who were COVID-19 positive after this date (post-U09.9 period).

The presence of long COVID-19–related ICD-10 codes was identified via a data-driven process using EHR data from 10 health care systems at the 4CE [19]. Initial steps consisted of extracting longitudinal codified features such as ICD-10 codes and mapping these codified features to phecodes for new onset of conditions after COVID-19 infection. Phecodes are a curated grouping of ICD-10 codes used to analyze EHR data characterizing specific clinical symptoms or diagnoses [20]. New onset conditions were defined as those that were not present before the initial COVID-19 infection. The conditions were selected such that patients with COVID-19 are associated with a higher risk of a new onset of the condition after adjusting for baseline confounders such as age, sex, self-reported race, and health care use. Marginal testing using a logistic regression framework was then performed to identify associated new onset conditions emerging 3 months after the initial infection. Conditions that passed marginal testing were then subject to conditional randomization analyses via distillation to robustly test whether a condition’s new onset is conditionally dependent on prior COVID-19 infection [19]. The Benjamini-Hochberg procedure was used to adjust for multiple comparisons [21]. Multimedia Appendix 1 [19,20] presents a list of the phecodes identified.

### Ethical Considerations

Exempt approval for this study was received by the Central Institutional Review Board at Veterans Affairs Boston Healthcare System (MVP000), BIDMC (2020P000565), and UPMC (STUDY20070095).

### Chart Review Approach

**Figure 2:**
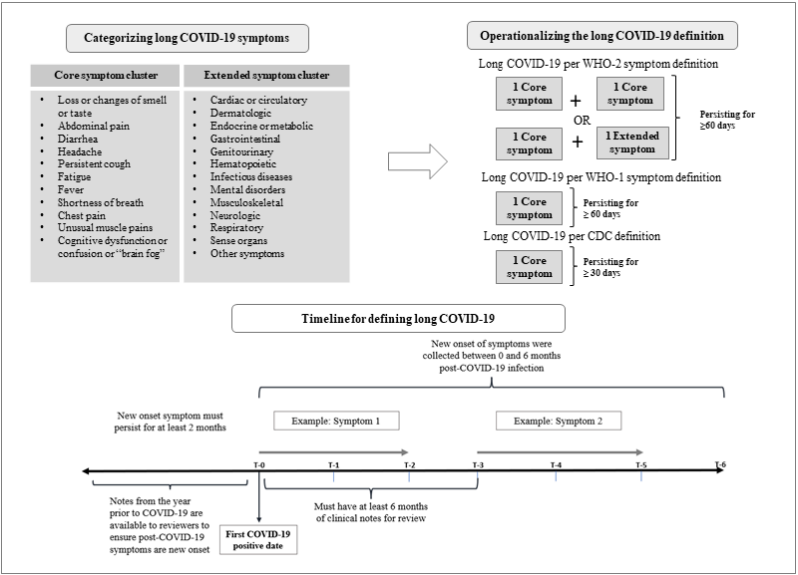
Chart review approach. CDC: Centers for Disease Control; WHO: World Health Organization.

The primary aim of our chart review was to operationalize the clinical case definition for long COVID-19 by the World Health Organization (WHO), with secondary aims to compare against a less stringent WHO definition and the Centers for Disease Control and Prevention (CDC) definition [3,22]. The chart review protocol (Multimedia Appendix 2) was developed at the VHA with guidance from 4CE subject matter experts to operationalize the WHO and CDC clinical case definitions. Long COVID-19 symptoms were identified from the WHO definition as well as through a literature review, and 11 commonly occurring symptoms among patients with long COVID-19 were classified into a “core” symptom cluster [23-26]. All other symptoms were classified into an “extended” symptom cluster based on their disease domain, which included cardiovascular, neurological, dermatological, musculoskeletal, digestive, and respiratory domains. For patients to be labeled as having long COVID-19 per the WHO definition during the chart review (reported here as “WHO-2”), at least 2 new onset symptoms after their COVID-19 infection were required (Figure 2). These could be either (1) two “core” symptoms or (2) one “core” and 1 “extended” cluster symptom, each of which must have persisted for 60 days or longer. All sampled patient charts had at least 6 months of clinical notes for review after the incident COVID-19 infection to allow appropriate assessment of symptoms. During chart review, all symptoms were collected based on their onset and duration of persistence for either 30 or 60 days (Multimedia Appendix 2) to allow evaluation against multiple long COVID-19 definitions. The less stringent WHO definition (reported here as “WHO-1”) was defined as a patient having just 1 core symptom persisting for at least 60 days or longer, and the CDC definition was defined as a patient having just 1 core symptom persisting for at least 30 days.

Reviewers had access to all clinical notes 1 year prior to the incident COVID-19 infection to determine baseline symptoms and conditions. Any symptoms present at the time of the incident COVID-19 infection or exacerbations of existing conditions were not considered new onset and thus not captured in the review. Chart reviewers were instructed to look for consistent mention or documentation of COVID-19-related symptoms in the notes and mark the duration of persistence (30 or 60 days). However, symptoms that waxed and waned over time were captured. At the VHA, a total of 500 patient charts were reviewed, and 200 patient charts were reviewed at each of the other 2 sites—BIDMC and UPMC.

### Characterizing the U09.9 ICD-10 Code

To characterize the use of the U09.9 ICD-10 code in clinical practice, the following three metrics were investigated: (1) the frequency of the U09.9 code used over time from October 2021 to September 2022, (2) the frequency of the U09.9 code used across Veterans Integrated Service Networks (VISNs; which are regional systems of care at the VHA), and (3) the time elapsed between COVID-19 diagnosis and U09.9 code assignment.

## Results

### Characteristics of Study Cohort

Demographics and cohort sizes across the health care systems varied; notably, patients in VHA who had a COVID-19 diagnosis between March 1, 2020, and December 31, 2021, were generally White and male veterans. Among those who were assigned a U09.9 code, the demographics were generally similar with a few notable exceptions (Table 1). At the BIDMC and UPMC, however, the demographics were different, with a higher proportion of female patients who were assigned the U09.9 code. We also observed across all 3 health care systems that a higher proportion of those assigned a U09.9 code had received at least 1 dose of a COVID-19 vaccine compared to the general population of patients who were COVID-19 positive.

**Figure 3:**
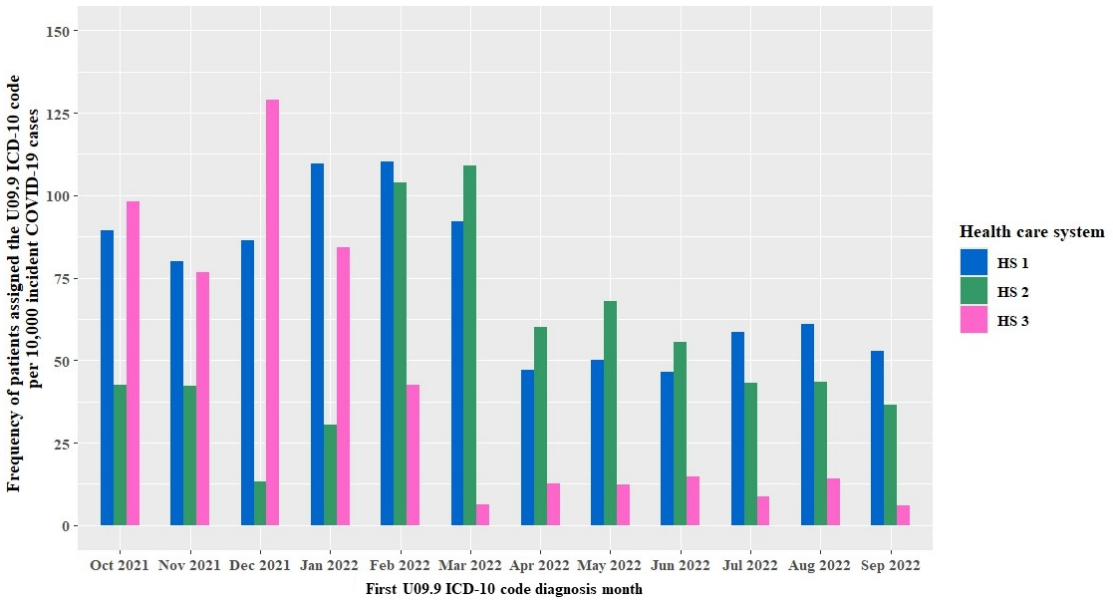
Frequency of a new U09.9 ICD-10 code assignment per 10,000 new COVID-19 cases in the last 12 months, not including the month of U09.9 diagnosis. HS: health care system; ICD-10: International Classification of Diseases, Tenth Revision.

**Figure 4:**
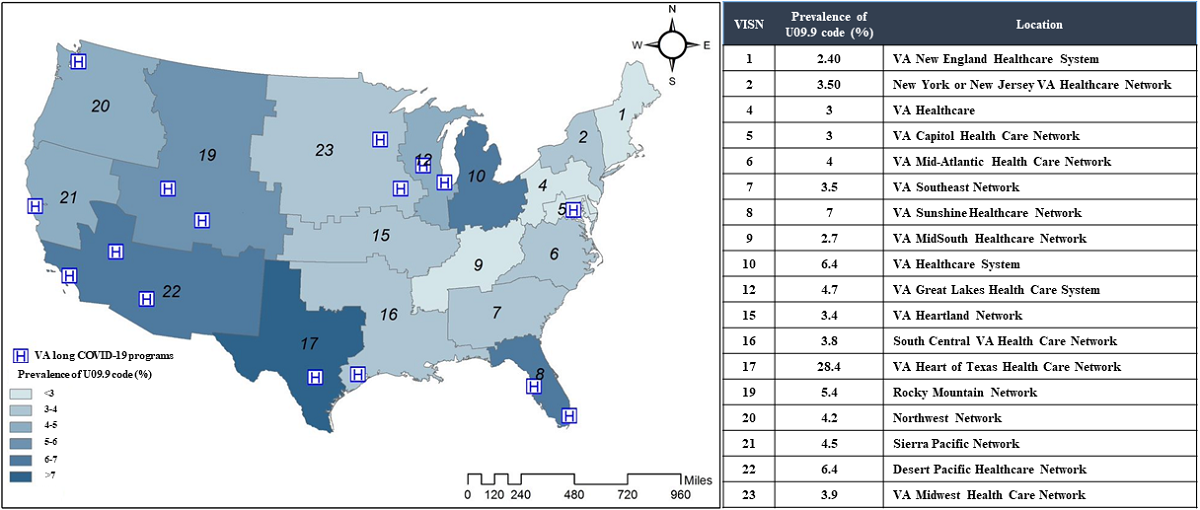
Prevalence of the U09.9 ICD-10 code by region. ICD-10: International Classification of Diseases, Tenth Revision; VA: Veterans Affairs; VISN: Veterans Integrated Service Networks.

We observed a substantial variation in the use of the U09.9 code to diagnose long COVID-19 over time and region. Figures 3 and 4 show the results of our characterizations of the U09.9 code use 12 months following its introduction in the United States on October 1, 2021. Figure 3 shows the frequency of the U09.9 code diagnosis per 10,000 new COVID-19 cases that occurred in the last 12 months from when they received the code. Results from the 3 health care systems were anonymized so as to provide an unbiased interpretation. The frequency of the U09.9 code used to diagnose long COVID-19 was highest from January to March 2022 at health care system 1, from February to March 2022 at health care system 2, and from December 2021 to January 2022 at health care system 3.

There were also large regional differences in the use of the U09.9 code across VHA health care system VISNs (Figure 4). VISN17 assigned the U09.9 code to 28.4% (6304/22,196) of all patients who received this code at the VHA, while VISN1 assigned the U09.9 code to just 2.4% (533/22,196) of all patients who received the code at the VHA.

**Table 1:** Patient demographics.

Demographics		Patient cohort and health care system									
		All patients who were COVID-19 positive^a^ from March 1, 2020, to December 31, 2021			All patients who were COVID-19 positive^a^ with a U09.9 ICD-10^b^ code				All patients who were COVID-19 positive^a^ with a new onset long COVID-19 feature		
		VHA^c^ (n=307,909)	BIDMC^d^ (n=30,294)	UPMC^e^ (n=147,653)	VHA (n=22,196)	BIDMC (n=164)	UPMC (n=6057)	VHA (n=294,302)		BIDMC (n=7245)	UPMC (n=92,120)
Age at incident COVID-19 diagnosis (years), mean (SD)		59.2 (16.1)	47.5 (20.5)	45.7 (23.8)	61.7 (15.1)	54.7 (13.9)	55.1 (17.2)	61.2 (15.7)		54.6 (18.4)	47.5 (24.1)
**Sex, n (%)**											
	Male	272,957 (88.7)	16,579 (54.7)	63,421 (43)	19,275 (86.8)	58 (35.4)	2219 (36.6)	260,055 (88.4)		2947 (40.67)	38,348 (41.6)
	Female	34,898 (11.3)	13,715 (45.3)	84,232 (57)	2921 (13.2)	106 (64.6)	3838 (63.4)	34,214 (11.6)		4298 (59.3)	53,772 (58.4)
**Race, n (%)**											
	American Indian or Alaska Native	3521 (1.1)	31 (0.1)	704 (0.5)	273 (1.2)	1 (0.6)	0 (0)	3282 (1.1)		8 (0.1)	493 (0.5)
	Asian	3326 (1.1)	1200 (4)	1303 (0.9)	249 (1.1)	4 (2.4)	70 (1.2)	3360 (1.1)		270 (3.7)	986 (1.1)
	Black or African American	69,067 (22.4)	5074 (16.7)	14,966 (10.1)	3621 (16.3)	29 (17.7)	880 (14.5)	69,037 (26.5)		1730 (23.9)	10,564 (11.5)
	Native Hawaiian or Pacific Islander	3189 (1)	21 (0.07)	35 (0.02)	239 (1.1)	0 (0)	0 (0)	3018 (1)		9 (0.1)	0 (0)
	White	208,457 (67.7)	8983 (29.7)	125,503 (85)	16,102 (72.5)	105 (64)	4965 (82)	197,237 (67)		3096 (42.7)	77,471 (84.1)
	Not reported	0 (0)	14,985 (49.5)	5141 (3.5)	0 (0)	25 (15.2)	142 (2.3)	0 (0)		2132 (29.4)	2606 (2.8)
**Vaccination status, n (%)**											
	Received at least 1 dose of COVID-19 vaccine	199,235 (64.7)	9649 (31.9)	58,244 (39.4)	15,295 (68.9)	112 (68.3)	3521 (58.1)	203,569 (69.2)		4470 (61.7)	41,271 (44.8)
	Did not receive at least 1 dose of COVID-19 vaccine	104,907 (34.1)	20,645 (68.1)	89,409 (60.6)	6684 (30.9)	52 (31.7)	2536 (41.9)	87,569 (29.8)		2775 (38.3)	50,849 (55.2)

^a^COVID-19 positive is defined as a patient having either a U07.1 ICD-10 code or a documented positive polymerase chain reaction test.

^b^ICD-10: International Classification of Diseases, Tenth Revision.

^c^VHA: Veterans Health Administration.

^d^BIDMC: Beth Israel Deaconess Medical Center.

^e^UPMC: University of Pittsburgh Medical Center.

### Chart Review Results

Chart review at the VHA was conducted by 2 clinical reviewers (M Maripuri and JH) with a 1% overlap and an interrater reliability of 80%. At the BIDMC and UPMC, a chart review was conducted by 1 clinical reviewer (BB-J and MJS, respectively). The most common symptoms identified during chart review among patients with long COVID-19 as per the WHO-2 definition were shortness of breath, fatigue, cough, and loss of smell or taste from the core symptom cluster. Among the extended symptom clusters, we most commonly saw symptoms across the cardiovascular, gastrointestinal, neurological, and respiratory disease domains.

**Figure 5:**
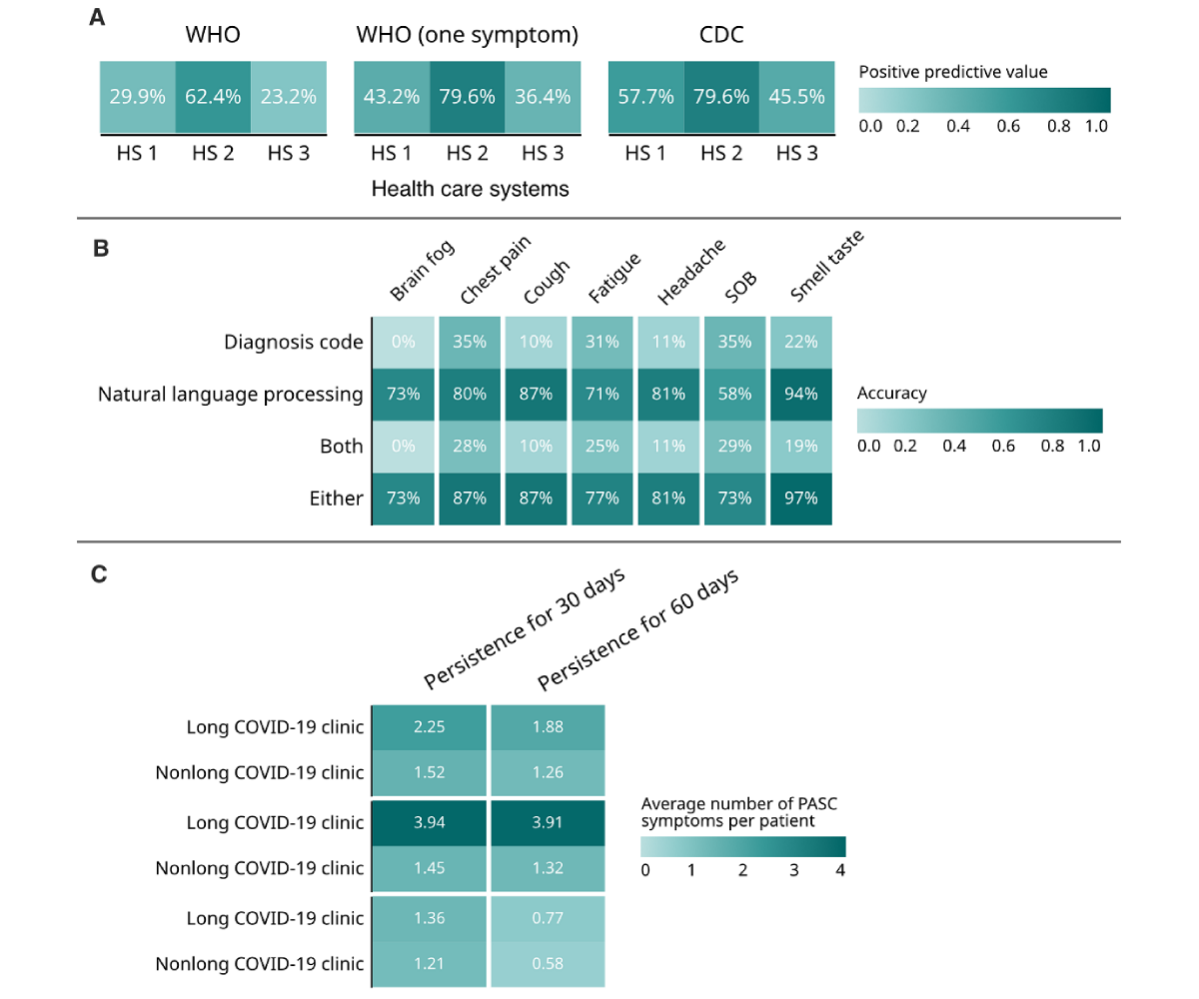
Comparison of results among all sites. (A) Predictive values of ICD-10 U09.9 code for various clinical definitions of long COVID-19. (B) Capture of PASC symptoms using diagnosis codes and natural language processing data. (C) Average number of new onset PASC symptoms in long COVID-19 clinic patients. CDC: Centers for Disease Control; HS: health care system; ICD-10: International Classification of Diseases, Tenth Revision; PASC: postacute sequelae of SARS-CoV-2; SOB: shortness of breath; WHO: World Health Organization.

Chart review was performed on a total of 900 patients infected with COVID-19 across 3 health care systems. These 3 institutions were anonymized to provide an unbiased interpretation of the results. The performance of the U09.9 code was evaluated by calculating the positive predictive value (PPV) or the probability of having long COVID-19 (as defined by chart review classification) among the patients who had the U09.9 code. When using the WHO-2 definition to define long COVID-19, the PPV was 29.8% at health care system 1 and 62.4% and 23.2% at health care systems 2 and 3, respectively (Figure 5). However, when we consider the WHO-1 and the CDC definitions, the PPV of long COVID-19 was higher at all 3 health care systems, but at health care system 2, the PPV was slightly higher for the WHO-1 definition and remained the same for the CDC definition (Figure 5).

We also evaluated PPV in the sample of patients with new onset long COVID-19 features. Using the WHO-2 definition, the PPV was 7% at health care system 1 and 6.7% and 3% at health care systems 2 and 3, respectively.

Weighted sensitivity calculations were used due to the nonrandom nature of our labeled set in relation to the overall cohort. Our approach involved a random sampling of patients with the U09.9 code, while those without this code were intentionally downsampled. Applying standard sensitivity calculations to such a biased labeled set could lead to misleading results. To counteract this, weighted sensitivity was used, which assigns a specific sampling weight to each case in our labeled data set. This weight is calculated to be inversely proportional to the likelihood of a case’s inclusion in the sample. By integrating this weighting system, we ensure that each case’s contribution to the sensitivity analysis accurately mirrors its representation in the full cohort, thereby yielding more representative and reliable results. The overall performance of the U09.9 code based on the WHO-2 definition resulted in a weighted sensitivity of 15% at health care system 1 and 4.9% and 19.1% at health care systems 2 and 3, respectively.

Additionally, at the VHA, we looked at the prevalence of long COVID-19 among patients with the U09.9 ICD-10 code across different time periods based on their first COVID-19 infection date. From the chart reviewed cohort, 44.6% (50/112) had long COVID-19 from the pre-U09.9 period and 22.7% (53/233) from the post-U09.9 period at the VHA. The PPV of patients with long COVID-19 in the pre-U09.9 period is higher, as many of these patients were backcoded.

Through our chart review, we observed that patients were given the U09.9 code over a wide range of time from fewer than 29 days to over 365 days following their initial COVID-19 infection. Most patients did not have persisting symptoms after acute infection, and waxing and waning of symptoms were frequently observed.

## Discussion

### Principal Results

This study provides a comprehensive, multicenter evaluation of the U09.9 code against proposed clinical case definitions for long COVID-19. It is also one of the first studies to apply a clinical case definition to EHR data using a chart review approach. The use of EHR data allowed evaluation of the U09.9 code across multiple health care systems nationwide. The availability of ample clinical notes enabled reviewers to ascertain whether observed symptoms after COVID-19 infection were truly new onset and to evaluate the duration of new symptoms, which are critical components of case definitions for long COVID-19. Another strength of this study was that we evaluated both the WHO and CDC clinical case definitions for long COVID-19 since one universal definition is not currently available. Our symptom collection approach (Multimedia Appendix 2) captured discrete symptoms by the duration of 30 or 60 days, which allowed for multiple case definitions to be applied.

There were large variations in the accuracy of the use of the U09.9 code for long COVID-19. We observed that 1 center had a much higher predictive value for patients with long COVID-19 among the U09.9 cohort across all definitions than the other 2 health care systems. This health care system also had the highest average number of new onset symptoms among patients seen in long COVID-19 clinics. The U09.9 code assignment at this health care system could have been more accurate due to a higher proportion of patients being seen at long COVID-19 clinics.

In a recent publication on this work, we also evaluated the capture of long COVID-19 symptoms using ICD-10 codes and natural language processing (NLP) data [14]. The Narrative Information Linear Extraction NLP tool was used to extract relevant concept unique identifiers that were manually mapped to each of the long COVID-19 symptoms we studied [27]. Among the chart-reviewed patients with the U09.9 code, we then identified the ones who had the various symptoms through the chart review. We then assessed what proportion of them with the symptom (ie, brain fog) had a corresponding ICD-10 or concept unique identifier mention of the concept and calculated the proportion of patients with the NLP or codified data capture. We shared the findings from this evaluation in Figure 5B. The performance of NLP was significantly better for all the commonly occurring long COVID-19 symptoms. The accuracy of using either a diagnosis code or NLP had the best results with 97% accuracy for loss of smell or taste and 87% accuracy for chest pain and cough.

### Limitations

There were several limitations to this study. The cohort at the VHA had a higher proportion of male patients who were generally older and predominantly White. Incident COVID-19 infection was required for inclusion in the chart review, and it is possible that patients had an infection outside of the health care systems that was not recorded in the EHR. Patients may have also had symptoms that were not reported at health care system visits. The variation in the number of long COVID-19 clinics across regions may have led to a differential capture of symptoms for those patients who were seen at long COVID-19 clinics versus those who were seen by their primary care providers. We observed that symptoms among these patients were well documented as most long COVID-19 clinics have a specific template for evaluating and capturing COVID-19 symptoms [28]. In some instances, it was difficult to assess whether a symptom was truly new onset due to COVID-19 infection or a result of underlying health conditions noted at baseline. While the WHO definition has been in use since 2021, long COVID-19 is still an evolving disease, and the case definition may change over time as the condition is further characterized. We also faced some challenges in optimizing a heterogenous and sparse data capture within the EHR systems.

### Comparison With Prior Work

The use of the U09.9 code has been described in several cohorts. The National Institutes of Health’s National COVID Cohort Collaborative (N3C) reported on the growing use of U09.9 from October 2021 through January 2022 in a nationwide cohort of 21,072 patients with the code [10]. However, the N3C did not require patients in the cohort to have a positive COVID-19 test to evaluate the use of the U09.9 code, and 37.2% (n=12,550) of patients did not have a COVID-19 index date. McGrath et al [12] also reported increasing use of the U09.9 code in the months following its release in the nationwide HealthVerity cohort of 56,143 patients with a U09.9 code that included children younger than 18 years of age. Similar to the N3C, this cohort did not require a COVID-19 positive test for evaluation of the U09.9 code, and only 70.4% (n=8879) had a documented COVID-19 infection. Of the patients who were COVID-19 positive with U09.9, the median time from infection to U09.9 diagnosis was 56 (21-200) days. A study in Sweden by Bygdell et al [11] reported 10,196 patients with the U09.9 code. They also found that 2% of the population who were COVID-19 positive in the 2 largest regions of Sweden had U09.9 at least 28 days after infection.

### Conclusions

Our findings suggest that the U09.9 code should be used judiciously in EHR-based studies of long COVID-19. Given the low PPV of the U09.9 code, its use as a proxy for long COVID-19 is not recommended. However, the sensitivity of the code makes it useful for identifying patients who may have long COVID-19 and thus require further clinical evaluation.

This was one of the initial efforts toward validating long COVID-19 against a clinical case definition and the U09.9 code through a chart review on a nationwide cohort. The chart review approach developed at the VHA can be implemented at other EHR systems to further evaluate the utility and performance of the U09.9 code. Further efforts to develop a more refined and reproducible phenotyping algorithm for long COVID-19 using NLP are underway, using the chart review labels from our study for algorithm training and development.

## Appendix

Multimedia Appendix 1 Selected postacute sequelae of SARS-CoV-2 feature phecodes.

Multimedia Appendix 2 Chart review protocol.

## Abbreviations

4CE: Consortium for Clinical Characterization of COVID-19 by Electronic Health Record
BIDMC: Beth Israel Deaconess Medical Center
CDC: Centers for Disease Control and Prevention
EHR: electronic health record
ICD-10: International Classification of Diseases, Tenth Revision
N3C: National COVID Cohort Collaborative
NLP: natural language processing
PPV: positive predictive value
UPMC: University of Pittsburg Medical Center
VHA: Veterans Health Administration
VISN: Veterans Integrated Service Networks
WHO: World Health Organization

## References

[1] Soriano JB, Murthy S, Marshall JC, Relan P, Diaz JV, WHO Clinical Case Definition Working Group on Post-COVID-19 Condition (2022). A clinical case definition of post-COVID-19 condition by a Delphi consensus. Lancet Infect Dis.

[2] Overview: COVID-19 rapid guideline: managing the long-term effects of COVID-19: guidance. National Institute for Health and Care Excellence.

[3] Long COVID or post-COVID condition. Centers for Disease Control and Prevention.

[4] Fritsche LG, Jin W, Admon AJ, Mukherjee B (2023). Characterizing and predicting post-acute sequelae of SARS CoV-2 infection (PASC) in a large academic medical center in the US. J Clin Med.

[5] Pfaff ER, Girvin AT, Bennett TD, Bhatia A, Brooks IM, Deer RR, Dekermanjian JP, Jolley SE, Kahn MG, Kostka K, McMurry JA, Moffitt R, Walden A, Chute CG, Haendel MA (2022). Identifying who has long COVID in the USA: a machine learning approach using N3C data. Lancet Digit Health.

[6] Thompson EJ, Williams DM, Walker AJ, Mitchell RE, Niedzwiedz CL, Yang TC, Huggins CF, Kwong ASF, Silverwood RJ, Di Gessa G, Bowyer RCE, Northstone K, Hou B, Green MJ, Dodgeon B, Doores KJ, Duncan EL, Williams FMK, Steptoe A, Porteous DJ, McEachan RRC, Tomlinson L, Goldacre B, Patalay P, Ploubidis GB, Katikireddi SV, Tilling K, Rentsch CT, Timpson NJ, Chaturvedi N, Steves CJ (2022). Long COVID burden and risk factors in 10 UK longitudinal studies and electronic health records. Nat Commun.

[7] Wulf Hanson S, Abbafati C, Aerts JG, Al-Aly Z, Ashbaugh C, Ballouz T, Blyuss O, Bobkova P, Bonsel G, Borzakova S, Global Burden of Disease Long COVID Collaborators (2022). Estimated global proportions of individuals with persistent fatigue, cognitive, and respiratory symptom clusters following symptomatic COVID-19 in 2020 and 2021. JAMA.

[8] Xie Y, Bowe B, Al-Aly Z (2021). Burdens of post-acute sequelae of COVID-19 by severity of acute infection, demographics and health status. Nat Commun.

[9] Al-Aly Z, Xie Y, Bowe B (2021). High-dimensional characterization of post-acute sequelae of COVID-19. Nature.

[10] Pfaff ER, Madlock-Brown C, Baratta JM, Bhatia A, Davis H, Girvin A, Hill E, Kelly E, Kostka K, Loomba J, McMurry JA, Wong R, Bennett TD, Moffitt R, Chute CG, Haendel M (2023). Coding long COVID: characterizing a new disease through an ICD-10 lens. BMC Med.

[11] Bygdell M, Leach S, Lundberg L, Gyll D, Martikainen J, Santosa A, Li H, Gisslén Magnus, Nyberg F (2023). A comprehensive characterization of patients diagnosed with post-COVID-19 condition in Sweden 16 months after the introduction of the International Classification of Diseases Tenth Revision diagnosis code (U09.9): a population-based cohort study. Int J Infect Dis.

[12] McGrath LJ, Scott AM, Surinach A, Chambers R, Benigno M, Malhotra D (2022). Use of the postacute sequelae of COVID-19 diagnosis code in routine clinical practice in the US. JAMA Netw Open.

[13] Ioannou GN, Baraff A, Fox A, Shahoumian T, Hickok A, O'Hare AM, Bohnert ASB, Boyko EJ, Maciejewski ML, Bowling CB, Viglianti E, Iwashyna TJ, Hynes DM (2022). Rates and factors associated with documentation of diagnostic codes for long COVID in the national veterans affairs health care system. JAMA Netw Open.

[14] Zhang HG, Honerlaw JP, Maripuri M, Samayamuthu MJ, Beaulieu-Jones BR, Baig HS, L'Yi S, Ho YL, Morris M, Panickan VA, Wang X, Weber GM, Liao KP, Visweswaran S, Tan BWQ, Yuan W, Gehlenborg N, Muralidhar S, Ramoni RB (2023). Potential pitfalls in the use of real-world data for studying long COVID. Nat Med.

[15] 4CE: consortium for clinical characterization of COVID-19 by EHR. Covidclinical.net.

[16] Veterans Health Administration. US Department of Veterans Affairs.

[17] By the numbers: UPMC facts and figures. UPMC.

[18] About Beth Israel Deaconess Medical Center. Beth Israel Deaconess Medical Center.

[19] Liu M, Katsevich E, Janson L, Ramdas A (2022). Fast and powerful conditional randomization testing via distillation. Biometrika.

[20] Denny JC, Bastarache L, Ritchie MD, Carroll RJ, Zink R, Mosley JD, Field JR, Pulley JM, Ramirez AH, Bowton E, Basford MA, Carrell DS, Peissig PL, Kho AN, Pacheco JA, Rasmussen LV, Crosslin DR, Crane PK, Pathak J, Bielinski SJ, Pendergrass SA, Xu H, Hindorff LA, Li R, Manolio TA, Chute CG, Chisholm RL, Larson EB, Jarvik GP, Brilliant MH, McCarty CA, Kullo IJ, Haines JL, Crawford DC, Masys DR, Roden DM (2013). Systematic comparison of phenome-wide association study of electronic medical record data and genome-wide association study data. Nat Biotechnol.

[21] Benjamini Y, Hochberg Y (2018). Controlling the false discovery rate: a practical and powerful approach to multiple testing. J R Stat Soc.

[22] Post COVID-19 condition (long COVID). World Health Organization.

[23] Sudre CH, Murray B, Varsavsky T, Graham MS, Penfold RS, Bowyer RC, Pujol JC, Klaser K, Antonelli M, Canas LS, Molteni E, Modat M, Jorge Cardoso M, May A, Ganesh S, Davies R, Nguyen LH, Drew DA, Astley CM, Joshi AD, Merino J, Tsereteli N, Fall T, Gomez MF, Duncan EL, Menni C, Williams FMK, Franks PW, Chan AT, Wolf J, Ourselin S, Spector T, Steves CJ (2021). Attributes and predictors of long COVID. Nat Med.

[24] Cervia C, Zurbuchen Y, Taeschler P, Ballouz T, Menges D, Hasler S, Adamo S, Raeber ME, Bächli Esther, Rudiger A, Stüssi-Helbling Melina, Huber LC, Nilsson J, Held U, Puhan MA, Boyman O (2022). Immunoglobulin signature predicts risk of post-acute COVID-19 syndrome. Nat Commun.

[25] Lambert N, El-Azab SA, Ramrakhiani NS, Barisano A, Yu L, Taylor K, Esperança Á, Mendiola C, Downs CA, Abrahim HL, Hughes T, Rahmani AM, Borelli JL, Chakraborty R, Pinto MD, Survivor Corps (2024). The other COVID-19 survivors: timing, duration, and health impact of post-acute sequelae of SARS-CoV-2 infection. J Clin Nurs.

[26] Su Y, Yuan D, Chen DG, Ng RH, Wang K, Choi J, Li S, Hong S, Zhang R, Xie J, Kornilov SA, Scherler K, Pavlovitch-Bedzyk AJ, Dong S, Lausted C, Lee I, Fallen S, Dai CL, Baloni P, Smith B, Duvvuri VR, Anderson KG, Li J, Yang F, Duncombe CJ, McCulloch DJ, Rostomily C, Troisch P, Zhou J, Mackay S, DeGottardi Q, May DH, Taniguchi R, Gittelman RM, Klinger M, Snyder TM, Roper R, Wojciechowska G, Murray K, Edmark R, Evans S, Jones L, Zhou Y, Rowen L, Liu R, Chour W, Algren HA, Berrington WR, Wallick JA, Cochran RA, Micikas ME (2022). Multiple early factors anticipate post-acute COVID-19 sequelae. Cell.

[27] Sheng Yu, Tianrun Cai, Tianxi Cai (2019). NILE: fast natural language processing for electronic health records. ArXiv.

[28] Gustavson AM, Eaton TL, Schapira RM, Iwashyna TJ, Adly M, Purnell A (2024). Approaches to long COVID care: the Veterans Health Administration experience in 2021. BMJ Mil Health.

